# Structure of native photosystem II assembly intermediate from *Chlamydomonas reinhardtii*

**DOI:** 10.3389/fpls.2023.1334608

**Published:** 2024-01-23

**Authors:** Mariia Fadeeva, Daniel Klaiman, Eaazhisai Kandiah, Nathan Nelson

**Affiliations:** ^1^ The George S. Wise Faculty of Life Sciences, Department of Biochemistry and Molecular Biology, Tel Aviv University, Tel Aviv, Israel; ^2^ CM01 Beamline, European Synchrotron Radiation Facility (ESRF), Grenoble, France

**Keywords:** photosystem II, photosynthesis, biogenesis of PSII, assembly intermediate, structure, CryoEM, assembly factor, *Chlamydomonas reinhardtii*

## Abstract

*Chlamydomonas reinhardtii* Photosystem II (PSII) is a dimer consisting of at least 13 nuclear-encoded and four chloroplast-encoded protein subunits that collectively function as a sunlight-driven oxidoreductase. In this study, we present the inaugural structure of a green alga PSII assembly intermediate (pre-PSII-int). This intermediate was isolated from chloroplast membranes of the temperature-sensitive mutant TSP4, cultivated for 14 hours at a non-permissive temperature. The assembly state comprises a monomer containing subunits A, B, C, D, E, F, H, I, K, and two novel assembly factors, Psb1 and Psb2. Psb1 is identified as a novel transmembrane helix located adjacent to PsbE and PsbF (cytochrome b559). The absence of PsbJ, typically found in mature PSII close to this position, indicates that Psb1 functions as an assembly factor. Psb2 is an eukaryotic homolog of the cyanobacterial assembly factor Psb27. The presence of iron, coupled with the absence of Q_A_, Q_B_, and the manganese cluster, implies a protective mechanism against photodamage and provides insights into the intricate assembly process.

## 1 Introduction

PSII is a significant protein-pigment complex integrated into the photosynthetic membranes of chloroplasts and cyanobacteria. Its primary role is to serve as a light-driven water-plastoquinone oxidoreductase. This supercomplex comprises numerous subunits, and its assembly requires the involvement of numerous assembly factors. These factors play vital roles in ensuring its proper construction, assembly, maintenance, and the removal of damaged units (Mulo et al., 2008; Bricker et al., 2012; Komenda et al., 2012; Vinyard et al., 2022).

The assembly of PSII is a stepwise process that necessitates various assembly factors, some of which are conserved from cyanobacteria to higher plants. The steps leading to the assembly of PSII core complexes have been extensively studied, primarily in cyanobacteria (Lu, 2016; Spaniol et al., 2022). While some of these steps have been confirmed in plants and green algae, not all have been observed. It is hypothesized that PSII assembly initiates with the synthesis of the α- and β-subunits (PsbE and PsbF) of cytochrome b559, which are present in etiolated chloroplasts along with a limited amount of D2 (Müller and Eichacker, 1999; Komenda et al., 2004). There is a suggestion that D1 first interacts with PsbI, followed by their assembly with the D2-cytochrome b559 complex into the reaction center (RC) (Dobáková et al., 2009). Subsequently, the low-molecular-mass subunits PsbH, PsbL, PsbM, PsbR, PsbT, PsbX, and PsbY join to form the RC47 intermediate (Boehm et al., 2012). The addition of CP43 with the small subunits PsbK, PsbZ, and Psb27 leads to the formation of PSII monomers (Sugimoto and Takahashi, 2003; Rokka et al., 2005; Boehm et al., 2011).

In chloroplasts, during photoactivation, the Mn4CaO5 cluster becomes associated with the lumenal side of PSII monomers, followed by the binding of the PsbO, PsbP, and PsbQ proteins (Bricker et al., 2012). Upon the dimerization of PSII monomers and subsequent attachment of LHCII trimers, the assembly process is finalized (Tokutsu et al., 2012). The ultimate Chlamydomonas PSII structure comprises up to 46 subunits (Sheng et al., 2021), which closely resembles the more comprehensive supercomplex identified in *Chlorella ohadii* (Fadeeva et al., 2023).

Due to the substantial complexity of the PSII supercomplex and the multitude of potential assembly intermediates, capturing and isolating these intermediates in a pure state and in sufficient quantities for structural determination is exceedingly challenging, particularly in eukaryotes. Eukaryotes, being difficult to target for mutagenesis, add to this challenge. As a result, much of the structural investigation has been conducted using cyanobacterial mutants with inhibited PSII assembly processes at various stages (Xiao et al., 2021; Yu et al., 2021; Zabret et al., 2021; Zhao et al., 2023).

To capture and elucidate the structure of the eukaryotic PSII assembly intermediate, the *Chlamydomonas reinhardtii* mutant TSP4 was employed. This mutant features a single amino acid substitution in PsbO, resulting in the entire PSII complex becoming temperature-sensitive (Bayro-Kaiser and Nelson, 2016; Bayro-Kaiser and Nelson, 2020).

## 2 Material and methods

### 2.1 Purification of the PSII intermediate

The C. reinhardtii strain TSP4 was cultivated in 8 liters of TAP media for 2 days at 18°C under continuous illumination (50 µmol m^-2^ s^-1^) until it reached a cell density of OD730 = 0.7-0.8. Subsequently, the culture was harvested by centrifugation at 900 g for 5 minutes, followed by resuspension in fresh 8 liters of TAP media preheated to 38.5°C. The culture was allowed to adapt for 14 hours at 38.5°C under continuous illumination (50 µmol m^-2^ s^-1^). Following adaptation, the culture was again harvested by centrifugation at 900 g for 5 minutes. The resulting cell pellet was resuspended and washed in 200 ml of cell buffer (composed of 50 mM Hepes at pH 7.5, 300 mM sucrose, and 5 mM MgCl2). Subsequently, the cells were pelleted again by centrifugation at 4,300 g for 5 minutes and resuspended in 50 ml of ice-cold PSII buffer (comprising 25 mM MES-NaOH at pH 6.0, 200 mM sucrose, 1 M glycine betaine, 10% glycerol, 1 mM MgCl2, and 10 mM NaCl).

To further process the cells, they were resuspended in 50 ml of PSII buffer containing 1 µM pepstatin, 1 mM benzamidine, and 1 mM PMSF. Cell disruption was achieved using an Avestin^®^ EmulsiFlex-C3 electric motor, using two cycles at 1500-2000 psi. The resulting lysate was then clarified by centrifugation for 5 minutes at 10,000 g. Finally, the membranes present in the supernatant were pelleted using ultracentrifugation (utilizing a Ti-70 rotor, 42,000 rpm for 40 minutes) and resuspended in PSII buffer to achieve a chlorophyll concentration of 2.0 mg/ml.

N-Dodecyl β-D-maltoside (β-DDM, Affymetrix™) and n-octyl-D-glucopyranoside (OG Anatrace) were added dropwise from a 10% stock solution to achieve a final concentration of 2.5% for each of them. The suspension was gently mixed manually a few times and then shaken gently on ice for 25 minutes to facilitate solubilization. After solubilization, the insoluble material was removed using an SS-34 rotor at 12,000 g for 10 minutes. The resulting supernatant was applied onto a sucrose gradient, ranging from 5% to 30% (approximately 1.9 mg of chlorophyll per tube) in a buffer containing 20 mM MES-NaOH at pH 6.0, 0.2% β-DDM, and 0.1% n-octyl-D-glucopyranoside (OG). This mixture was then centrifuged in an SW-40 rotor at 37,000 rpm for 16 hours. The resulting gradient was fractionated based on previous experiments, and Fraction 3 (**Supplementary Figure 1**) was chosen for further processing. It was diluted with a buffer containing 20 mM MES-NaOH at pH 6.0, 1 mM MgCl2, and 0.1% β-DDM to decrease the sucrose concentration and subsequently concentrated using VivaSpin^®^ 20 (MWCO 30,000 Da). The concentrated fraction was then applied onto a 15-45% sucrose gradient with the same dilution buffer and centrifuged in an SW-60 rotor at 48,000 rpm for 16 hours.

The resulting gradient bands were collected and subjected to analysis using SDS-PAGE and Western Blotting (**Supplementary Figure 2**). The medium fraction, which contained the PSII subunits of interest, was diluted with a buffer containing 20 mM MES-NaOH at pH 6.0, 1 mM MgCl2, and 0.1% β-DM to reduce the sucrose concentration. This diluted sample was then concentrated using VivaSpin^®^ 20 (MWCO 50,000 Da). The concentrated fraction obtained was applied onto a 15-45% sucrose gradient with the same buffer and centrifuged in an SW-60 rotor at 48,000 rpm for 16 hours. The resulting gradient bands were collected and analyzed using SDS-PAGE and Western Blotting (as indicated in **Supplementary Figure 3**). Fraction MII was chosen for structural analysis and was diluted with a buffer containing 20 mM MES-NaOH at pH 6.0, 1 mM MgCl2, and 0.1% β-DM to reduce the sucrose concentration. Subsequently, it was concentrated using VivaSpin^®^ 20 (MWCO 50,000 Da) to achieve a chlorophyll concentration of 0.5 mg/ml.

### 2.2 SDS-PAGE and immunoblotting

Isolated thylakoids and purified complexes were dissociated using sodium dodecyl sulfate (SDS) sample buffer. We employed SDS-polyacrylamide gel electrophoresis (PAGE) with a 17% gel to separate the proteins, and subsequently transferred these proteins to a nitrocellulose membrane using a wet transfer method (utilizing Bio-Rad Mini-PROTEAN Tetra Cell and Mini Trans-Blot^®^ Cell), following the manufacturer’s instructions. The amount loaded corresponded to 0.5 or 1.0 µg of chlorophyll.

All antibodies utilized in this study were purchased from Agrisera. The antibodies used, along with their respective catalog numbers, are as follows: PsbA (AS11 1786), PsbB (AS04 038), PsbC (AS11 1787), PsbD (AS06 146), PsbO (AS06 142-33), CP29 (Lhcb4) homolog, *Chlamydomonas* (AS06 117), CP26 (Lhcb5) homolog, *Chlamydomonas* (AS09 407), PsaA (AS06 172), PsaC (AS10 939), PsaD (AS09 461).

### 2.3 Cryo-EM data collection, processing, and model building

3 µl of pre-PSII-1 at a concentration of 0.5 mg/ml chlorophyll were applied onto glow-discharged holey carbon grids and vitrified for cryo-EM structural analysis using the Leica-EM-GP instrument (with a 3-second blot at 20°C and 100% humidity). Data were collected on a 300-kV Titan Krios G3 Microscope by Thermo Fisher Scientific (ESRF, Grenoble, France). Movies were recorded in counting mode at a magnification of ×105,000, yielding a calibrated pixel size of 0.85 Å. A total of 25,758 micrographs were obtained with a total dose of 45 e/Å2 (as shown in **Supplementary Figure 4A**).

Both heterogeneous and homogeneous refinement processes were conducted for pre-PSII-1. Subsequently, the particles (numbering 6,408,350) were converted into a Star file format and imported into RELION 3.1.1. The particles were re-extracted (un-binned) and processed in RELION using a box size of 400 pixels for pre-PSII-1. A 3D classification with 2 classes was performed (**Supplementary Figure 4C**). From this, a class containing 951,575 high-quality particles was chosen and subjected to 3D refinement, resulting in an overall resolution of 3.1 Å (**Supplementary Figure 4D**).

Further analysis included CTF refinement, additional 3D refinement, Bayesian polishing, followed by another round of CTF refinement for pre-PSII-1. This culminated in another 3D refinement, achieving an overall resolution of 2.9 Å for pre-PSII-1.

## 3 Results and discussion

**Figure 1:**
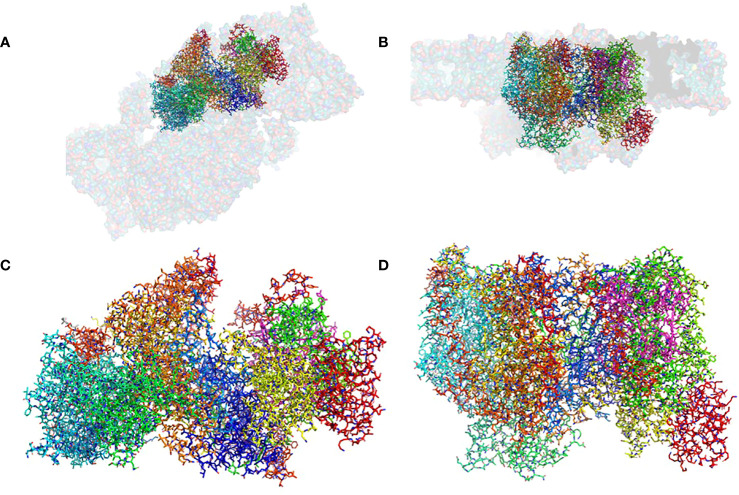
The structure of the pre-PSII-1: **(A)** A view from the lumen at an 80% transparent overlay of the *C*. *reinhardtii* PSII structure (PDB 6KAC). **(B)** A side view from the membrane level at an 80% transparent overlay of the PSII structure (PDB 6KAC). **(C)** A view from the lumen. **(D)** A side view from the membrane level.

Earlier work involved generating and characterizing the *C. reinhardtii* mutant TSP4 (Bayro-Kaiser and Nelson, 2016; Bayro-Kaiser and Nelson, 2020). At the non-permissive temperature, the unstable mutant subunit PsbO causes the degradation of PSII. Simultaneously, the newly formed PSII complex undergoes maturation and stabilization processes, potentially involving the repair of damaged PSII units. Due to the instability of the mutant complex, the accumulation of fully assembled PSII is impeded, resulting in the accumulation of the most stable assembly intermediate (**Figure 1**). Notably, the relative amounts of PSI subunits remain unchanged (**Supplementary Figure 5**). This phenomenon, as indicated in **Supplementary Figure 6**, differs from other temperature-sensitive *C. reinhardtii* mutants, which often display an overaccumulation of fully assembled, active PSII complexes, leading to a decrease in the amount of PSI fraction. For example, in the cytochrome *b6f* temperature-sensitive mutant grown at the non-permissive temperature (Schwartz et al., 2023), an opposite pattern is observed, demonstrating an accumulation of fully assembled active PSII complexes (as depicted in **Supplementary Figure 6**). Therefore, this distinctive property of temperature-dependent PSII degradation appears to be specific to this particular mutant exhibiting temperature-sensitive photosynthesis (Bayro-Kaiser and Nelson, 2016).

The mutant strain was cultivated, processed, and the assembly intermediate was purified using standard procedures, as detailed in the Methods section. A total of 25,758 movies were collected, and reconstructions were generated at 2.9 Å (as shown in **Supplementary Figure 4**). The map dimensions of the particles (140 x 110 Å) indicated a distinct protein composition with missing subunits (**Supplementary Figures 4B, C**). The well-defined density enabled the construction of an atomic model representing a subcomplex composed of nine subunits: PsbA, PsbB, PsbC, PsbD, PsbE, PsbF, PsbH, PsbI, PsbK, Psb1, and Psb2, designated as pre-PSII-1 (**Figure 1** and **Supplementary Figure 4**).

**Figure 2:**
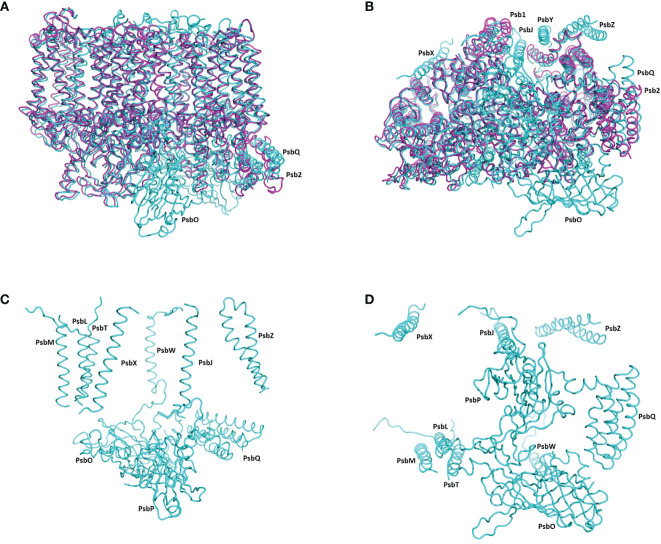
The structure of pre-PSII-1 superposed on the core subunits of *C*. *reinhardtii* PSII. **(A)** A side view from the membrane level; pre-PSII-1 in magenta and PSII in cyane. **(B)** A view from the lumen; pre-PSII-1magenta and PSII in cyane. **(C)** A side view from the membrane level on the structure of subunits missing in pre-PSII-1. **(D)** A view from the lumen on the structure of subunits missing in pre-PSII-1.

**Figure 3:**
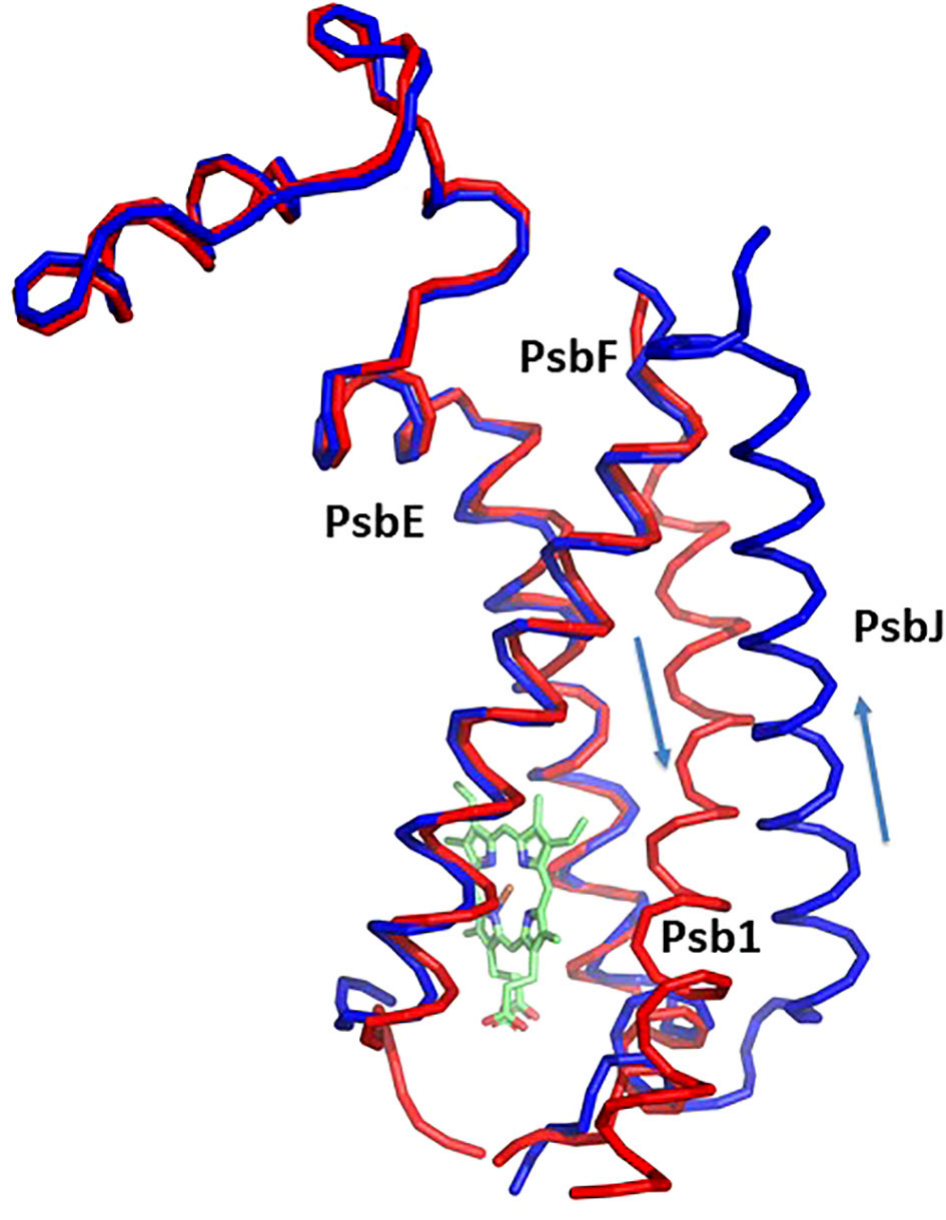
Structure of cytochrom *b*_559_ and the chaperon Psb1. Pre-PSII-1 in red superposed on intact PSII in blue. The helices directions of Psb1 and PsbJ are indicated.

Most of the subunits within pre-PSII-1 retained their positions as observed in the intact PSII (PDB 6KAC) (**Figures 2A, B**). **Figures 2C, D** show side and lumen views of subunits missing in pre-PSII-1 in comparison with the intact PSII (PDB 6KAC). A slight displacement was noted for PsbE and PsbF, which are part of the cytochrome *b559*. PsbJ, positioned between the cytochrome and the majority of the other subunits, was absent. Within this gap, a distinct transmembrane helix named Psb1 was clearly identified. Through examination of the *C. reinhardtii* genome, the chloroplast-encoded gene psb1 was identified, reasonably matching the density map (Maul et al., 2002) (**Figures 2B**, **3** and **Supplementary Figure 4F**). As a result, it is proposed that Psb1 serves as an assembly factor at this stage of PSII assembly. At this stage, the pre-PSII-1 assembly intermediate awaits the introduction of PsbJ, replacing Psb1, to mature this segment of the complex. It is suggested that the absence of Psb1 shifts the equilibrium toward the degradation of the assembly intermediate reported in this study. This suggestion aligns with the effect of plant PsbJ gene targeting inactivation, which did not affect PSII assembly but rendered it unstable and light-sensitive (Hager et al., 2002).

Ycf12 was recognized earlier as a possible PSII subunit (Kashino et al., 2007; Takasaka et al., 2009). Early structural analyses identified Ycf12 as PsbY (PDB 3AOB, 6DHE, and 4PBU), positioned at the periphery of the PSII complex. PsbY shows no sequence relation to the protein Psb1 identified in this study (Kawakami et al., 2009; PDB 3A0B). The pre-PSII-1 assembly intermediate does not contain LPA2 or rubredoxin, which might function in catalytic capacities (García-Cerdán et al., 2019; Cecchin et al., 2021; Spaniol et al., 2022; Calderon et al., 2023).

PSII assembly is facilitated by auxiliary factors that transiently bind to discrete assembly intermediates and are not incorporated into the final complex. Many of these auxiliary factors are conserved between cyanobacteria and chloroplasts, although not all of them share this conservation (Nixon et al., 2010; Bricker and Frankel, 2011; Nickelsen and Rengstl, 2013). Current understanding of the assembly of cyanobacterial PSII is enriched by a wealth of genetic, biochemical, and structural information (Xiao et al., 2021; Zabret et al., 2021; Zhao et al., 2023). High-resolution structures of several cyanobacterial assembly intermediates have been resolved, allowing for comparative analysis with the present *Chlamydomonas* pre-PSII-1 structure.

**Figure 4:**
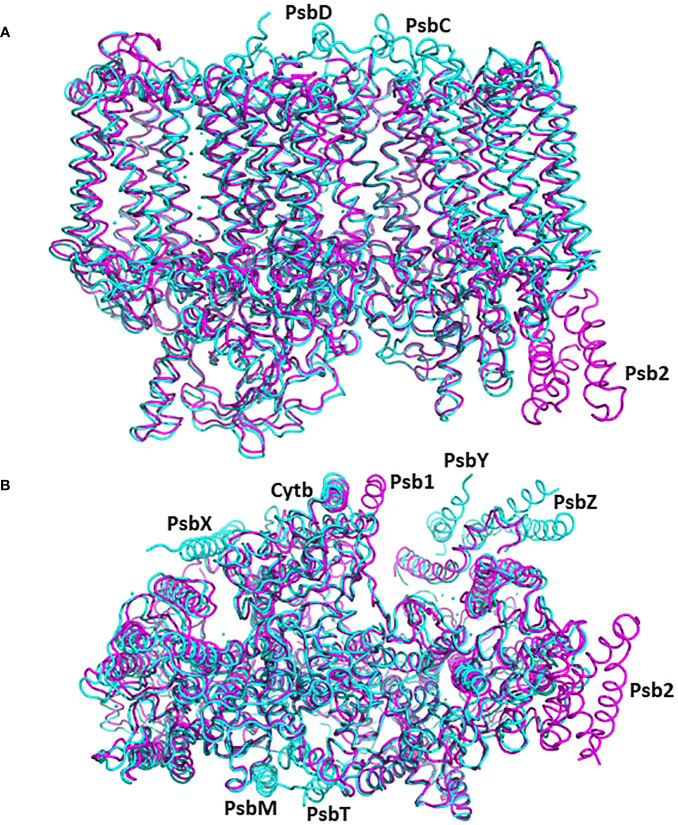
The structure of pre-PSII-1 superposed on the cyanobacterial assembly intermediate PSII-M (PDB 7NHO). The pre-PSII-1 is in magenta and PSII-M in cyan. **(A)** A side view from the membrane level. **(B)** A view from the lumen.

In this comparative analysis, three well-established structures of cyanobacterial intermediates (PSII-M 7NHO, PSII-I 7NHP, and PSII-I’ 7NHQ; detailed in Zabret et al., 2021) were utilized to compare the various subunits of the C. reinhardtii pre-PSII-1. Superimposing the cyanobacterial assembly intermediate PSII-M onto the pre-PSII-1 revealed primary distinctions between procaryote and eukaryote PSII assembly intermediates (as depicted in **Figure 4**). The peripheral subunits PsbM, T, X, Y, and Z are exclusively found in PSII-M but are absent in pre-PSII-1. Conversely, the assembly factors Psb1 and Psb2 (homologous to the cyanobacterial Psb27) are present only in pre-PSII-1.

**Figure 5:**
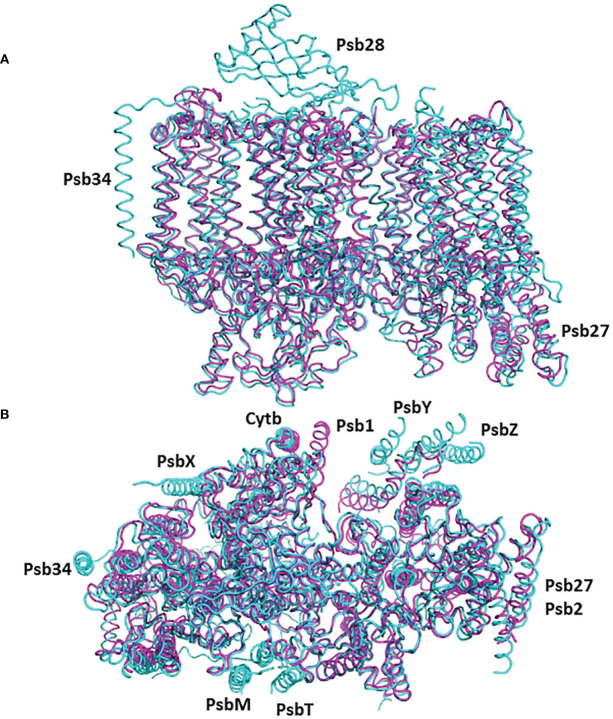
The structure of pre-PSII-1 superposed on the cyanobacterial assembly intermediate PSII-I (PDB 7NHP). The pre-PSII-1 is in magenta ans PSII-I in cyan. **(A)** A side view from the membrane level. **(B)** A view from the lumen.

The cyanobacterial assembly intermediate PSII-I shares similar subunits with PSII-M, but it includes three additional assembly factors: Psb28, Psb27, and Psb34 (as illustrated in **Figure 5**). Among these factors, while Psb28 is absent in eukaryotes, Psb27 shows clear homology to Psb2 in C. reinhardtii pre-PSII-1 (**Supplementary Figure 7**). Although a Psb28 homolog, exhibiting a 59% sequence identity, is present in the *Chlamydomonas* genome and might function as an early auxiliary factor, it is not found in pre-PSII-1 (Dobáková et al., 2009).

**Figure 6:**
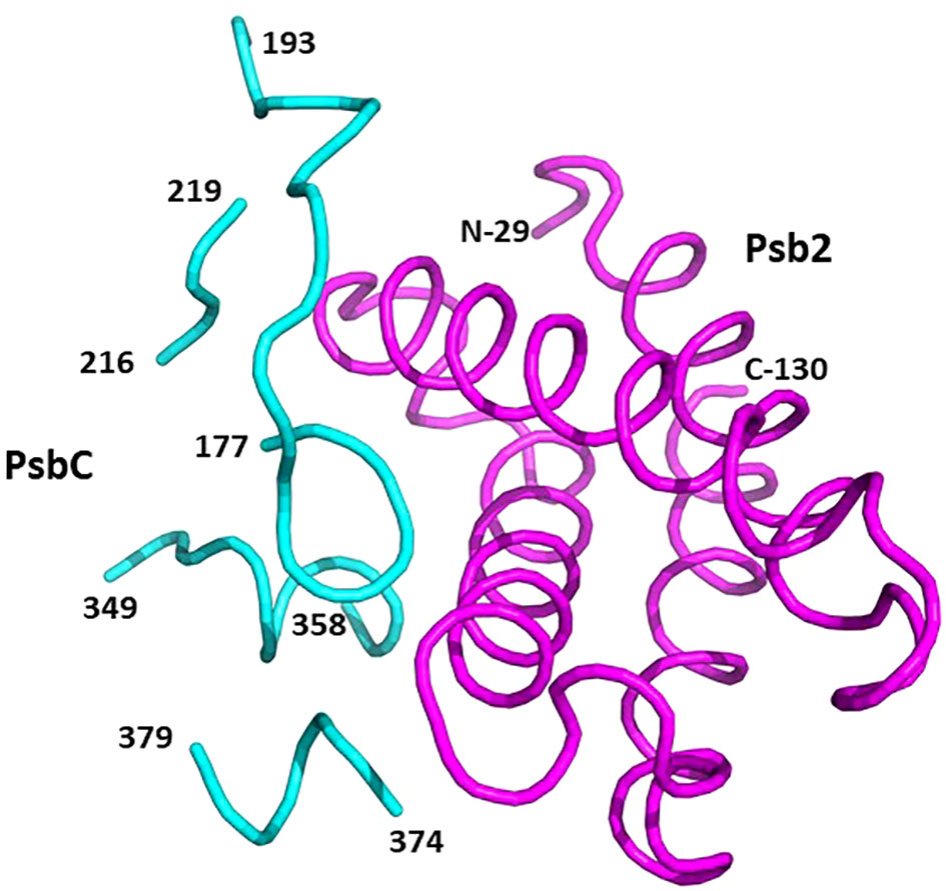
Binding sites on PsbC (CP43) for Psb2. Psb2 is in magenta and PsbC is in cyan. The numbers taken from the amino acid sequences of the respective subunits (PDB 8R2I).

**Figure 7:**
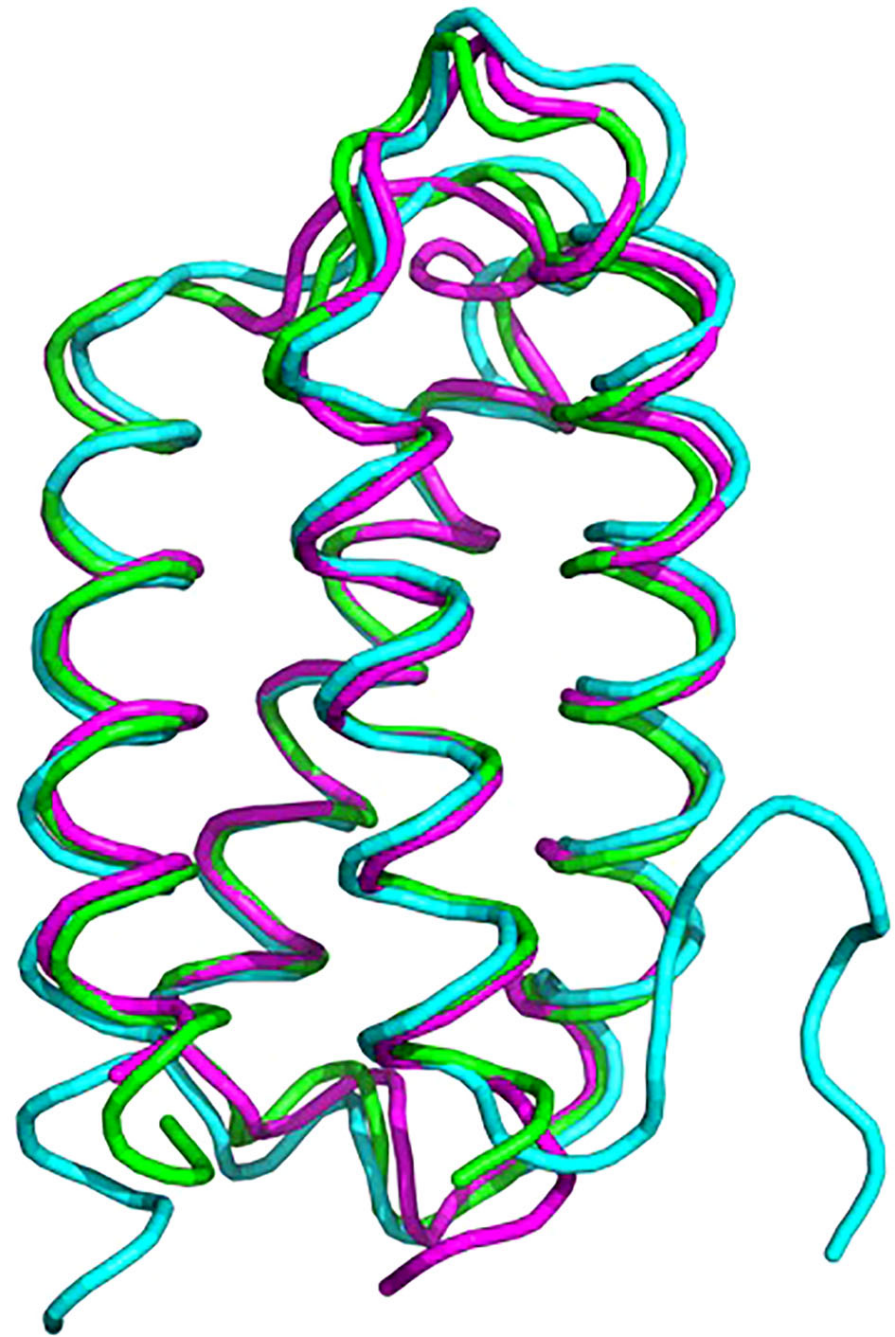
Superposition of proteins from *C. reinhardtii* Psb2 (magenta), cyanobacterial Psb27 (cyan) and plant (green). The plant structure is of Psb27 from *Arabidopsis thaliana* PDB 5x56, cyanobacterial structure is from *Thermosynechococcus* PDB 7NHP.

Psb2, composed of a four-helix bundle, is observed to interact with PsbC, suggesting a stabilizing role for pre-PSII-1 (**Figure 6**). An impressive structural conservation is noted among its homologous proteins from cyanobacteria and higher plants, such as Psb27 from Arabidopsis thaliana (Cheng et al., 2018; Xingxing et al., 2018) (**Figure 7** and **Supplementary Figure 7**).

**Figure 8:**
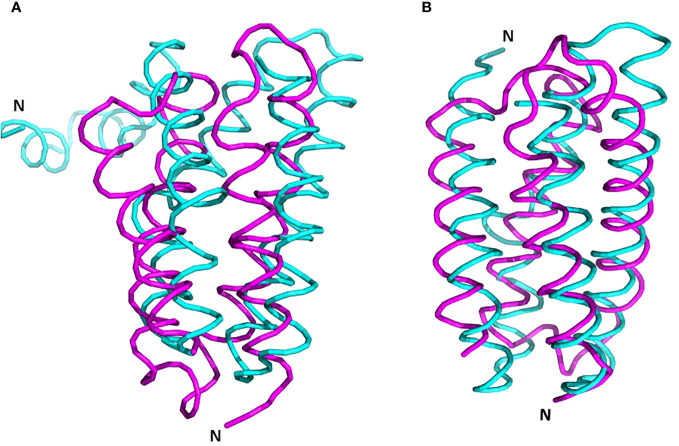
Superposition of PrePSII-1 Psb2 and PsbQ of mature PSII. **(A)** The position of Psb2 versus PsbQ where the structures of the native PSII and the structure of pre-PSII-int were aligned at PsbC. **(B)** The tow proteins were aliigned one on top the other. Psb2 in magenta and PsbQ in cyan. The N-terminus is indicated. PDB 6kac was used for the mature PSII.

It’s essential to note that a structurally similar component, the four-helix bundle PsbQ, is present in mature PSII, despite lacking sequence homology and exhibiting an opposite orientation (**Figure 8**, Burton-Smith et al., 2019; Fadeeva et al., 2023). During maturation, it is plausible that PsbQ substitutes Psb2 and acts as a template for the assembly of the extrinsic proteins PsbO and PsbP, thereby rendering its presence unnecessary in the mature PSII. This assumption gains support from its absence in several structures of intact PSII from various plants and algae (references: 5XNM - Su et al., 2017; 6KAD - Sheng et al., 2019; 6KAF - Shen et al., 2019; 8C29 - Opatikova et al., 2023; 7PI0 and 7PIW - Caspy et al., 2023).

The pre-PSII-1 assembly intermediate at the PSII acceptor side exhibits a distortion in the binding pocket of the mobile quinone (Q_B_) and includes the nonheme iron. In the map density towards the stromal surface, there’s a degree of poor resolution, but it contains extra density, suggesting a potential ligand for the non-heme iron, possibly glutamate. Notably, the absence of the Mn4CaO5 cluster on the lumen side is observed. These observations shed light on mechanisms that act as safeguards for PSII during its biogenesis, potentially serving to protect it from damage until water splitting is activated.

## Data availability statement

The raw data supporting the conclusions of this article will be made available by the authors, without undue reservation. The atomic coordinates of the prePSII-1 complex have been deposited in Protein Data Bank, with accession PDB 8R2I.

## Author contributions

NN: Conceptualization, Data curation, Funding acquisition, Investigation, Methodology, Project administration, Supervision, Writing – original draft, Writing – review & editing. MF: Conceptualization, Data curation, Formal analysis, Investigation, Methodology, Writing – review & editing. DK: Data curation, Formal analysis, Investigation, Software, Writing – review & editing. EK: Formal analysis, Investigation, Methodology, Software, Writing – review & editing.

## Supplementary material

Supplementary Figure 1 First step of PSII intermediate purification after membrane solubilization. **(A)** SW-40 gradient fractions collected for further analysis and purification; **(B)** SDS-PAGE of the collected fractions with control purified preparation of *C. reinhardtii* PSII, 0.5 µg Chl/well for each lane. On the grounds of the previous experiments and relative amounts of proteins in the marked area (location of CP47 and CP43 subunits on the gels) the fraction #3 was chosen for the next step.

Supplementary Figure 2 Second step of PSII intermediate purification. **(A)** SW-60 gradient fractions collected for further analysis and purification; **(B)** SDS-PAGE of the collected fractions with control purified preparation of *C. reinhardtii* PSII, 0.5 µg Chl/well for each lane. **(C)** Western-blot analysis of the collected fractions with control purified preparation of *C. reinhardtii* PSII, 0.5 µg Chl/well for each lane. Fraction M(medium), containing the highest amount of PSII core subunits, was chosen for the next purification step.

Supplementary Figure 3 Third step of PSII intermediate purification. **(A)** SW-60 gradient fractions collected for further analysis and purification; **(B)** SDS-PAGE of the collected fractions with control purified preparation of *C. reinhardtii* PSII, 0.5 µg Chl/well for each lane; **(C)** Western-blot analysis of the collected fractions with control purified preparation of *C. reinhardtii* PSII, 0.5 µg Chl/well for each lane. Fraction MII, containing the highest amount of PSII core subunits, and the least amount of PSI additions, was chosen for intermediate structural analysis; **(D)** Concentrated fraction MII, used for preparation of CryoEM samples and subsequent structural analysis, in comparison with full active PSII preparation isolated from TSP4 strain grown at permissive temperature.

Supplementary Figure 4 Cryo-EM data collection and processing scheme for unstacked PSII complex. **(A)** Sample micrograph collected for the PSII assembly intermediate dataset displaying PSII particles from multiple views; **(B)** 2D classes PSII assembly intermediate were created in RELION followed by 3D classification; **(C)** Best 3D classes with numbers and percentage of all chosen particles. These three classes gave in total 95.2% of all particles. Each of other seven 3D classes presented less the 1.5% and were considered insignificant. Only predominant class comprising 73.2% of particles was subjected to further refinement. **(D)** Final model of PSII assembly intermediate with a color-coded global resolution of 2.9 Å. **(E)** Fourier shell correlation (FSC) RELION postprocessing result marks a 2.9 Å resolution based on the 0.143 gold-standard. **(F)** Density map of Psb1.

Supplementary Figure 5 Characterization of PSI and PSII composition before and after cell growth under the non-permissive temperature. **(A)** SDS-PAGE of the TSP4 thylakoids, before heat treatment (0h) and after 17 hours of growth under conditions of non-permissive temperature 38.5°C, 0.5 µg chl/well for each lane; **(B, C)** Western-blot analysis of the same thylakoids with probing to subunits of PSII and PSI, respectively, to show changes in the relative changes of PSII and PSI amounts, 0.5 µg chl/well for each lane.

Supplementary Figure 6 Influence of single-amino acid temperature-sensitive mutation on the gradient appearance and relative content of PSII bands for the same amount of chlorophyll loaded. **(A)** Gradient for wild-type strain grown at permissive temperature; **(B)** gradient for TSP9 strain grown at non-permissive temperature; **(C)** SDS-PAGE of fractions collected from sample **(B)**. 1 – large form of PSII, 2 – small form of PSII, 3 – mostly PSI-containing band, upper thick band consist of LHS trimers and monomers.

Supplementary Figure 7 Alignment of representative Psb27 proteins. 1 - uncharacterized protein CHLRE_05g243800v5 [*Chlamydomonas reinhardtii*]. 2 - chain A of Photosystem II repair protein PSB27-H1; *Arabidopsis thaliana* Psb27. 3 - photosystem II protein Psb27 [*Thermosynechococcus vestitus*]. Identical amino acids are in red, amino acids with similar properties and structure are in blue.
