# Supplementary figures and images for: Structure of native photosystem II assembly intermediate from Chlamydomonas reinhardtii
Source: Front Plant Sci. 2024 Jan 23;14:1334608. doi: 10.3389/fpls.2023.1334608 (PMC10844431; doi:10.3389/fpls.2023.1334608)

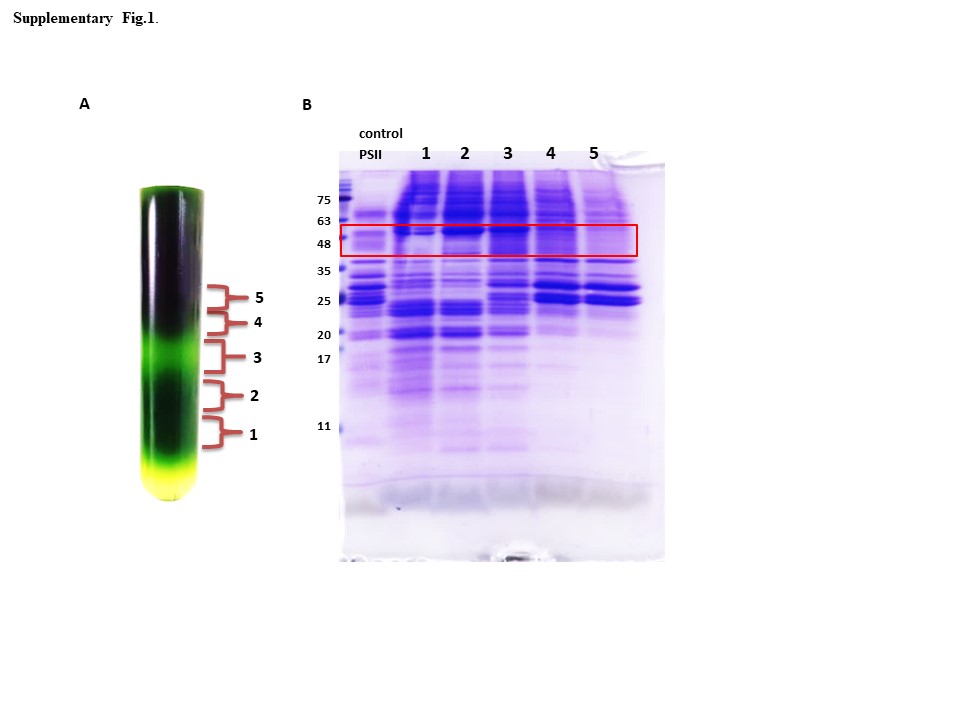

Supplement: Supplementary Figure 1 — First step of PSII intermediate purification after membrane solubilization. (A) SW-40 gradient fractions collected for further analysis and purification; (B) SDS-PAGE of the collected fractions with control purified preparation of C. reinhardtii PSII, 0.5 µg Chl/well for each lane. On the grounds of the previous experiments and relative amounts of proteins in the marked area (location of CP47 and CP43 subunits on the gels) the fraction #3 was chosen for the next step. [file Image_1.jpeg]

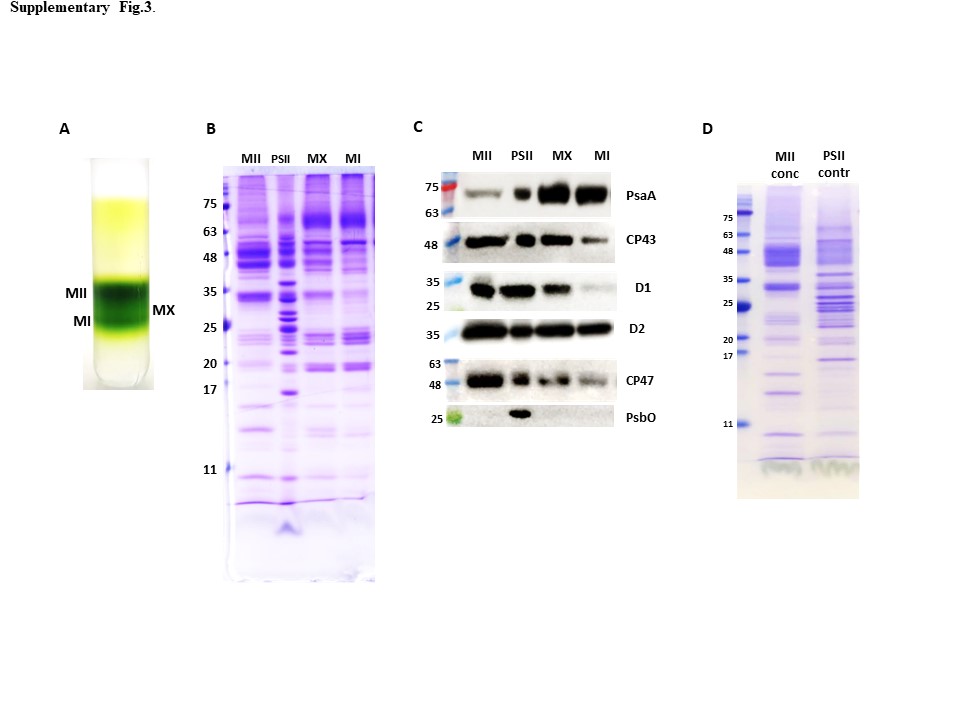

Supplement: Supplementary Figure 2 — Second step of PSII intermediate purification. (A) SW-60 gradient fractions collected for further analysis and purification; (B) SDS-PAGE of the collected fractions with control purified preparation of C. reinhardtii PSII, 0.5 µg Chl/well for each lane. (C) Western-blot analysis of the collected fractions with control purified preparation of C. reinhardtii PSII, 0.5 µg Chl/well for each lane. Fraction M(medium), containing the highest amount of PSII core subunits, was chosen for the next purification step. [file Image_2.jpeg]

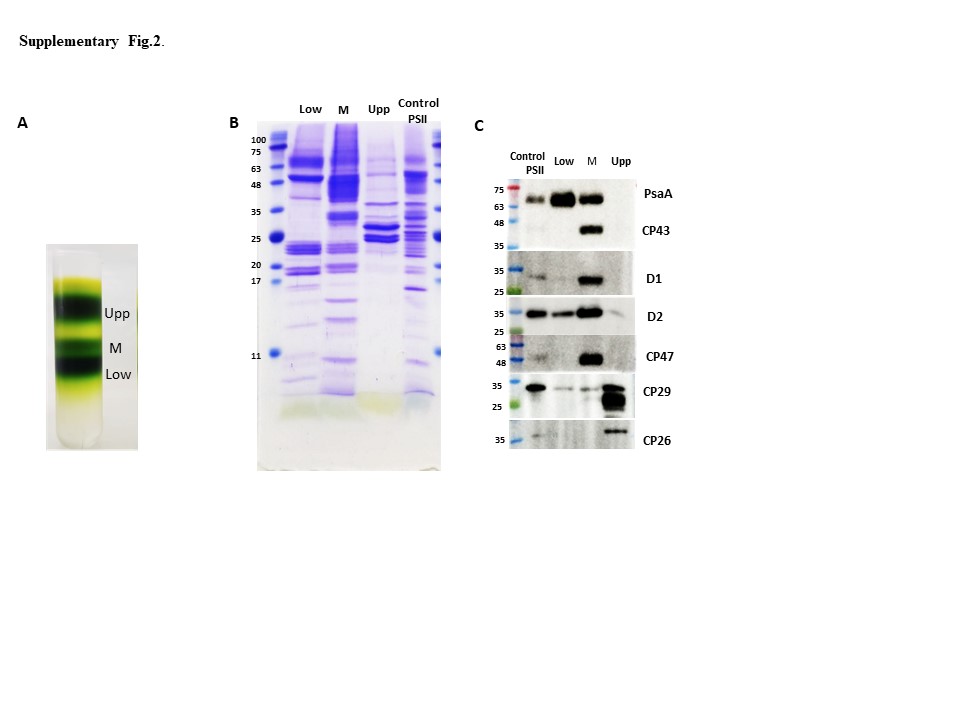

Supplement: Supplementary Figure 3 — Third step of PSII intermediate purification. (A) SW-60 gradient fractions collected for further analysis and purification; (B) SDS-PAGE of the collected fractions with control purified preparation of C. reinhardtii PSII, 0.5 µg Chl/well for each lane; (C) Western-blot analysis of the collected fractions with control purified preparation of C. reinhardtii PSII, 0.5 µg Chl/well for each lane. Fraction MII, containing the highest amount of PSII core subunits, and the least amount of PSI additions, was chosen for intermediate structural analysis; (D) Concentrated fraction MII, used for preparation of CryoEM samples and subsequent structural analysis, in comparison with full active PSII preparation isolated from TSP4 strain grown at permissive temperature. [file Image_3.jpeg]

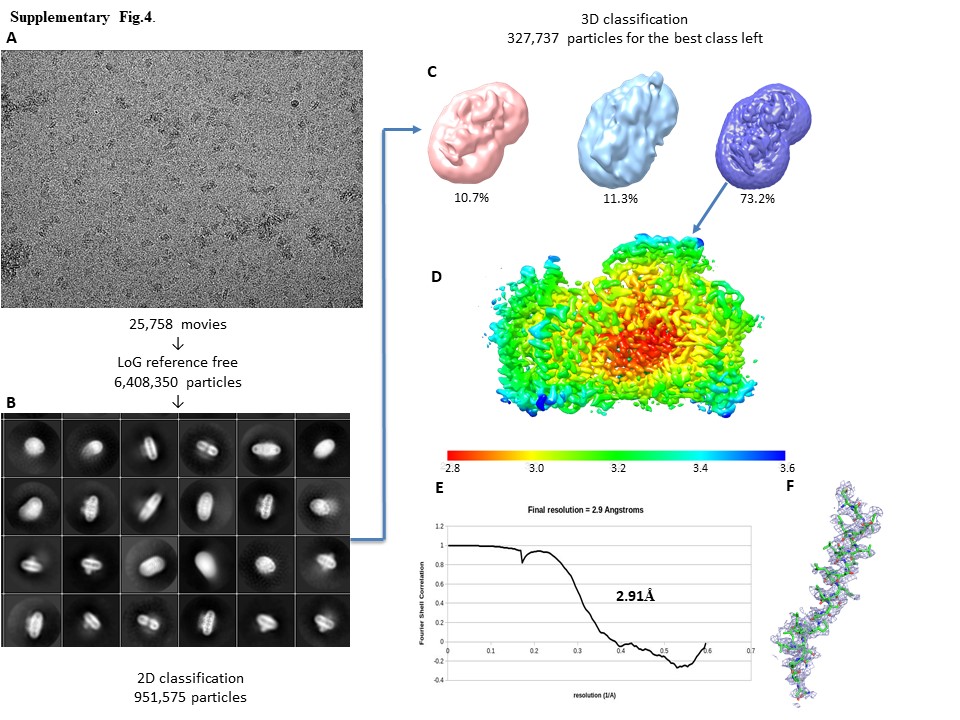

Supplement: Supplementary Figure 4 — Cryo-EM data collection and processing scheme for unstacked PSII complex. (A) Sample micrograph collected for the PSII assembly intermediate dataset displaying PSII particles from multiple views; (B) 2D classes PSII assembly intermediate were created in RELION followed by 3D classification; (C) Best 3D classes with numbers and percentage of all chosen particles. These three classes gave in total 95.2% of all particles. Each of other seven 3D classes presented less the 1.5% and were considered insignificant. Only predominant class comprising 73.2% of particles was subjected to further refinement. (D) Final model of PSII assembly intermediate with a color-coded global resolution of 2.9 Å. (E) Fourier shell correlation (FSC) RELION postprocessing result marks a 2.9 Å resolution based on the 0.143 gold-standard. (F) Density map of Psb1. [file Image_4.jpeg]

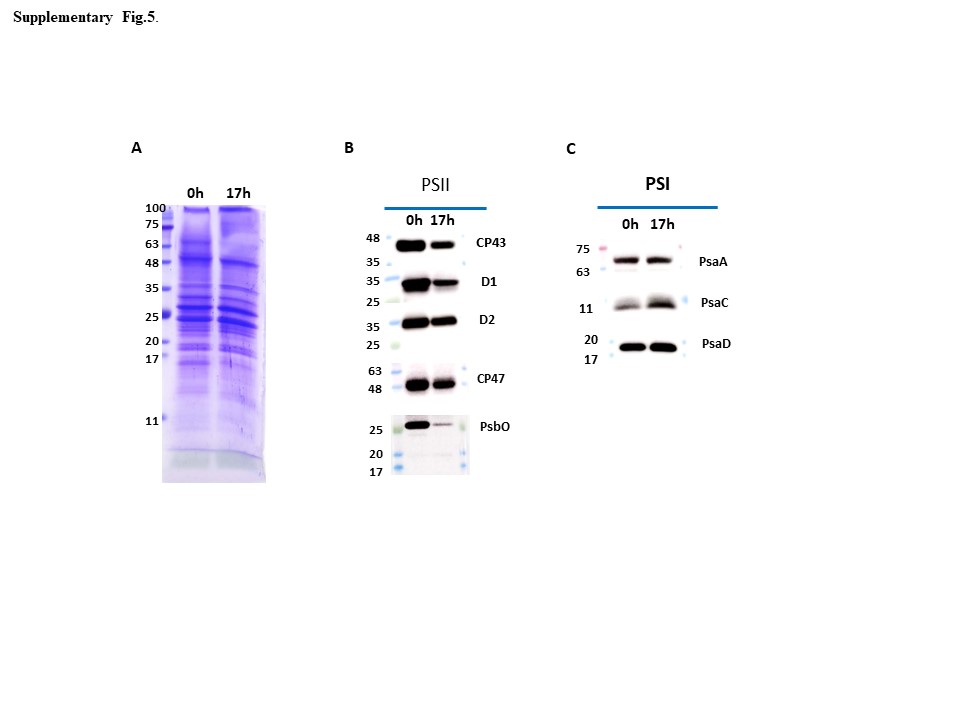

Supplement: Supplementary Figure 5 — Characterization of PSI and PSII composition before and after cell growth under the non-permissive temperature. (A) SDS-PAGE of the TSP4 thylakoids, before heat treatment (0h) and after 17 hours of growth under conditions of non-permissive temperature 38.5°C, 0.5 µg chl/well for each lane; (B, C) Western-blot analysis of the same thylakoids with probing to subunits of PSII and PSI, respectively, to show changes in the relative changes of PSII and PSI amounts, 0.5 µg chl/well for each lane. [file Image_5.jpeg]

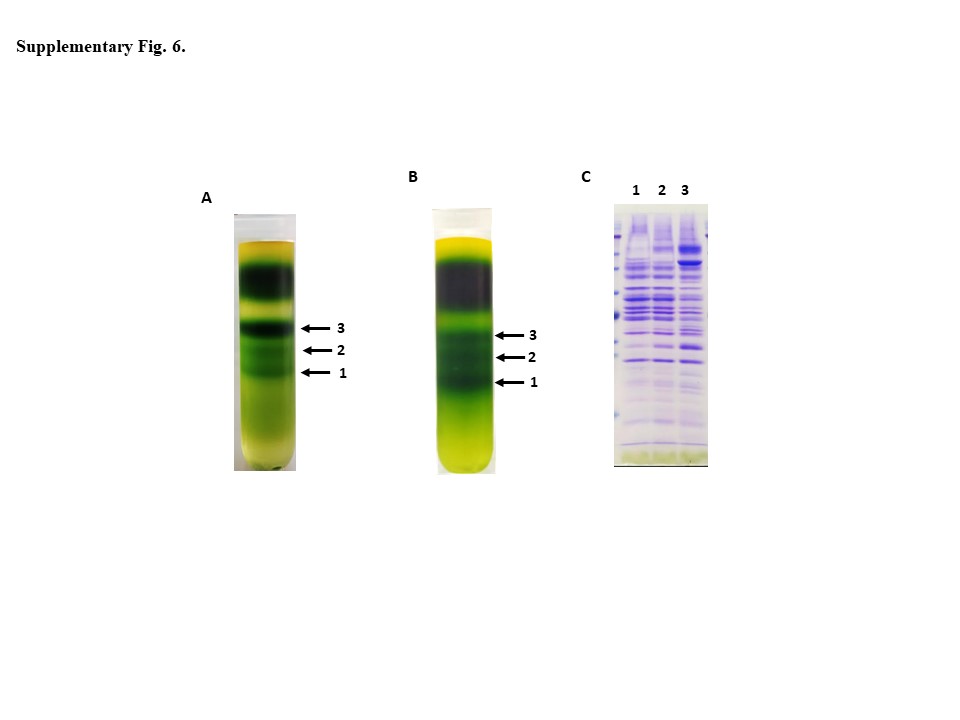

Supplement: Supplementary Figure 6 — Influence of single-amino acid temperature-sensitive mutation on the gradient appearance and relative content of PSII bands for the same amount of chlorophyll loaded. (A) Gradient for wild-type strain grown at permissive temperature; (B) gradient for TSP9 strain grown at non-permissive temperature; (C) SDS-PAGE of fractions collected from sample (B). 1 – large form of PSII, 2 – small form of PSII, 3 – mostly PSI-containing band, upper thick band consist of LHS trimers and monomers. [file Image_6.jpeg]

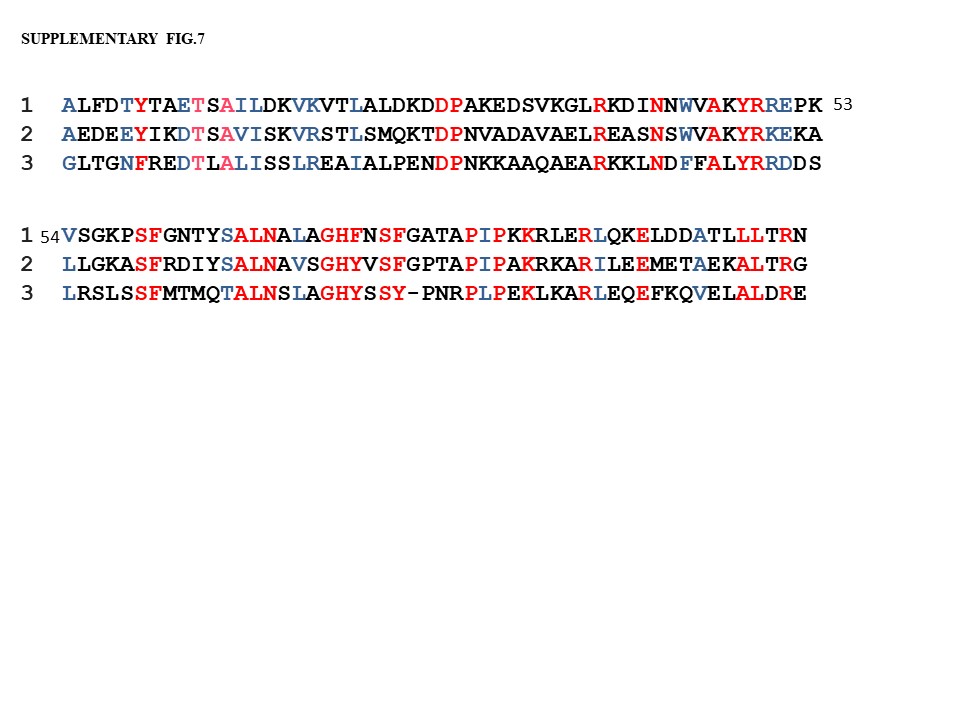

Supplement: Supplementary Figure 7 — Alignment of representative Psb27 proteins. 1 - uncharacterized protein CHLRE_05g243800v5 [Chlamydomonas reinhardtii]. 2 - chain A of Photosystem II repair protein PSB27-H1; Arabidopsis thaliana Psb27. 3 - photosystem II protein Psb27 [Thermosynechococcus vestitus]. Identical amino acids are in red, amino acids with similar properties and structure are in blue. [file Image_7.jpeg]
